# Polymeric Materials Obtained by Extrusion and Injection Molding from Lignocellulosic Agroindustrial Biomass

**DOI:** 10.3390/polym15204046

**Published:** 2023-10-10

**Authors:** Ada Pacheco, Arian Evangelista-Osorio, Katherine Gabriela Muchaypiña-Flores, Luis Alejandro Marzano-Barreda, Perla Paredes-Concepción, Heidy Palacin-Baldeón, Maicon Sérgio Nascimento Dos Santos, Marcus Vinícius Tres, Giovani Leone Zabot, Luis Olivera-Montenegro

**Affiliations:** 1 Bioprocesses and Biomass Conversion Research Group, Universidad San Ignacio de Loyola, La Molina 15024, Peru; ada.pacheco.gil@gmail.com (A.P.); arian.evangelista@usil.pe (A.E.-O.); katherine.muchaypina@usil.pe (K.G.M.-F.); lmarzano@usil.edu.pe (L.A.M.-B.); heidy.palacin@usil.pe (H.P.-B.); 2 Grupo de Ciencia, Tecnología e Innovación en Alimentos, Universidad San Ignacio de Loyola, La Molina 15024, Peru; pparedes@usil.edu.pe; 3 Laboratory of Agroindustrial Processes Engineering (LAPE), Federal University of Santa Maria, 1040 Sete de Setembro St., Center DC, Cachoeira do Sul, Santa Maria 96508-010, RS, Brazil; maiconsergions@gmail.com (M.S.N.D.S.); marcus.tres@ufsm.br (M.V.T.); giovani.zabot@ufsm.br (G.L.Z.)

**Keywords:** agroindustrial wastes, biomaterials, cellulose, lignocellulosic biomass

## Abstract

This review presents the advances in polymeric materials achieved by extrusion and injection molding from lignocellulosic agroindustrial biomass. Biomass, which is derived from agricultural and industrial waste, is a renewable and abundant feedstock that contains mainly cellulose, hemicellulose, and lignin. To improve the properties and functions of polymeric materials, cellulose is subjected to a variety of modifications. The most common modifications are surface modification, grafting, chemical procedures, and molecule chemical grafting. Injection molding and extrusion technologies are crucial in shaping and manufacturing polymer composites, with precise control over the process and material selection. Furthermore, injection molding involves four phases: plasticization, injection, cooling, and ejection, with a focus on energy efficiency. Fundamental aspects of an injection molding machine, such as the motor, hopper, heating units, nozzle, and clamping unit, are discussed. Extrusion technology, commonly used as a preliminary step to injection molding, presents challenges regarding fiber reinforcement and stress accumulation, while lignin-based polymeric materials are challenging due to their hydrophobicity. The diverse applications of these biodegradable materials include automotive industries, construction, food packaging, and various consumer goods. Polymeric materials are positioned to offer even bigger contributions to sustainable and eco-friendly solutions in the future, as research and development continues.

## 1. Introduction

The growing awareness of environmental challenges and the search for sustainable solutions have led to a critical evaluation of the way natural resources and waste are managed [1,2]. The continuous growth of the global population and the increasing demand for food and energy have made the effective management of agricultural and food waste a fundamental area of concern [3,4]. On an annual basis, a considerable quantity of agroindustrial wastes, arising from the production of food and crops, amasses on a global scale. This accumulation has adverse repercussions not only for the environment, but also for the global economy [5]. These residues not only represent a loss of valuable resources, but also cause increasing emissions of greenhouse gases, thus contributing to climate change [6]. Furthermore, at various stages across the food supply chain, from production to consumption, there is a disconcerting level of food loss and waste [7,8]. The food industry has embarked on a concerted endeavor to curtail food loss and waste, embracing strategies that champion the reevaluation of food waste. In this process, the concept of the circular economy has assumed a central role, advocating for the conversion of waste into valuable resources [9,10].

These waste materials, primarily composed of lignocellulosic biomass, can be efficiently converted into biopolymers [11,12]. Biomass, mainly comprising cellulose, hemicellulose, and lignin, necessitates pretreatment to reduce its refractory nature and enhance accessibility within its structure [13]. There are four types of pretreatment methods: physical (milling, extrusion, sonication, microwave, ultrasound, ozonolysis, and pyrolysis), chemical (alkali, dilute acid, ionic liquid, organic solvent, and oxidative delignification), physicochemical (CO_2_ explosion, steam explosion, hydrothermal, liquid hot water, and ammonia fiber explosion), and biological [14,15,16]. The characteristics of the feedstock, energy requirements, cost, and product recovery should be considered when choosing the pretreatment method [17].

Extrusion and injection molding are widely used manufacturing technologies in the plastics industry [18]. By leveraging this technology, biomass can be transformed into high-quality polymeric materials with desirable properties. Extrusion involves the continuous melting, mixing, and shaping of the biopolymers, while injection molding enables the precise and efficient formation of complex shapes through the injection of molten materials into molds [19].

The use of lignocellulosic agroindustrial biomass for polymeric materials offers numerous advantages, such as reduced dependence on polymers based on non-renewable fossil fuels, thereby promoting sustainability, and reducing the environmental impact [20]. In addition, this approach contributes to efficient waste management, reducing the burden on landfills [21].

Reinforced polymers, also called composites, are the union of two materials, a matrix, and a reinforcement, characterized by one being lightweight and the other strong [22]. The matrix can be polymeric, ceramic, or metallic, while the reinforcement can be fibers, particles, or laminates [23]. A challenge in the formation of composites is the coupling of the hydrophilic interfaces in the reinforcement and the hydrophobic interfaces in the polymeric matrix [24]. Fiber reinforcement is mainly composed of lignocellulosic mass. Agroindustrial wastes are increasingly used due to their low cost, biodegradability, improved properties, and composite quality [24].

This review delves into the latest innovations and research trends in the use of lignocellulosic biomass from agroindustrial wastes, with the purpose of developing polymeric materials using extrusion and injection molding technologies. Additionally, it provides an overview of agroindustrial biomass, its properties, pretreatment methods, and extrusion and injection molding processes. The review also underscores the wide-ranging industrial applications of these materials and outlines potential future developments.

## 2. Sources and Components of Lignocellulosic Agroindustrial Biomass

Lignocellulosic agroindustrial biomass, a highly renewable and cost-effective natural resource, is derived from agricultural residues (husks, bagasse, seeds, roots, leaves, stems, seed pods, and straw), food processing waste (peels, skin, shells, oil cakes, and egg waste), and forestry by-products [9,25]. The primary components of biomass are cellulose, hemicellulose, and lignin. The components can vary based on factors such as the type of biomass, location, climate, and harvesting season [12].

### 2.1. Cellulose

**Figure 1:**
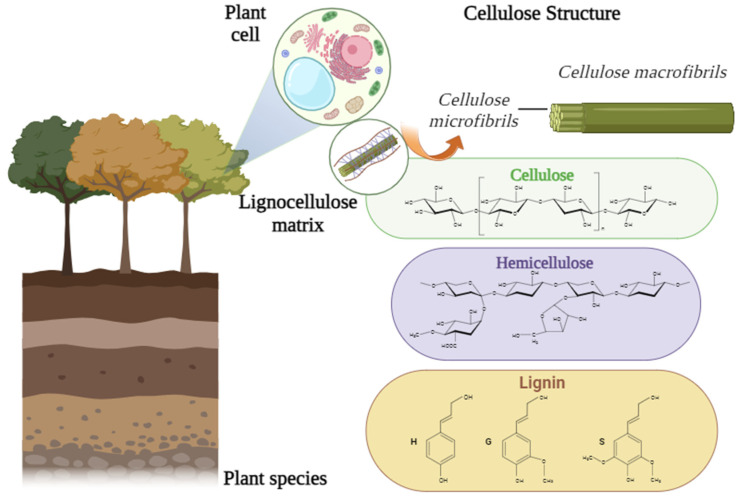
Matrix with cellulose macrofibrils and microfibrils intimately intertwined by the matrix of lignin and hemicellulose.

Cellulose (C_6_H_10_O_5_)_n_ is the most abundant renewable natural polymer in nature. In general, lignocellulose-based biomaterials have a large proportion of total cellulose content, highly interlaced by a significant amount of covalent bonds with high rigidity to form extremely strong and resilient components [26]. Generally, cellulose from algae is approximately 70 wt% and cellulose from plant-based materials ranges from 40 to 60 wt% [27]. Nonetheless, some studies indicate that the cellulose content in some plants, such as hop stems, can reach 70 wt%, which allows the substance to be widely used for biopolymer production and applications [28]. Spontaneously, cellulose molecules form large agglomerates that aggregate into microfibrils, which are constituents commonly called crystalline and amorphous zones [29]. The structure of the multiple components that comprise the complex are presented in Figure 1. The structure of cellulolytic chains is made up of cellulose microfibrils, intimately intertwined in complexes based on lignin and hemicellulose.

One of the main assertions about cellulose, and its stocked reserve of highly renewable and widely investigated organic constituents, is directed at the ease of obtaining the biopolymer. Expressive amounts of cellulose are easily verified in a series of plant species, marine algae, marine animals, bacteria, and vegetable residual biomass, which represent up to 50 wt% of the total weight of the biowaste [30]. Additionally, cellulose promotes high resistance in the plant cell wall, mainly due to the large number of glucose monomeric units covalently linked through β-1,4 glycosidic bonds [31].

Furthermore, other characteristics of the cellulose complex give rise to the recalcitrant characteristic of lignocellulose-based materials, such as the high crystalline performance of the matrix, a significant degree of polymerization (up to 10,000 units), and the presence of an intricate network of hydroxyl groups associated with intramolecular hydrogen bonds in cellulose [32]. The glucose-rich aggregate of hydroxyl compounds forms intertwined hydrogen bonds that provide resilience to the molecular structure and connect with neighboring particles to form a network of microfibrils. The hundreds of bonds that involve intermolecular and intramolecular hydrogen molecules and the intertwining of crystalline and non-crystalline zones are intimately responsible for the two-phase structure of cellulose, in which the regions of high crystallinity or cellulose nanocrystals (CNC) stand out [33]. Conversely, more susceptible molecular chains are called amorphous zones, which are easily degraded to obtain a highly soluble and reactive amorphous material. This performance promotes a drastic decline in solubility in liquid contents and an increase in resistance to molecular chain disfigurement by the action of water [34]. Additionally, cellulolytic chains include β-D-glucopyranose elements interconnected via β-(1,4)-glycosidic bonds. Cellulose holds up to 1400 D-glucose units directly disposed to structure microfibrils units, which are broadly grouped to configure cellulose fibrils, which are structured under a highly rigid and vigorous matrix, rich in cellulose and hemicellulose [35].

The diversity of applications of cellulose complexes is closely associated with a range of matrix dominances, such as low density, biodegradability, significant porosity, and improved physical and mechanical mechanisms [36]. Cellulose is easily obtained from natural sources, which corroborates its high accessibility, cost effectiveness, applicability, reduced or minimal toxicity, and biocompatibility [37]. The total cellulose content and the arrangement of the crystalline zones are dependent on the plant species and the lignocellulose content, which is directly associated with the resistance potential of the biomaterial and the difficulty of breaking the complex by the action of hydrolysis [38]. Furthermore, there is a diversity in the secondary structures derived from cellulose, or crystal arrangements, such as cellulose I, cellulose II, cellulose III, and cellulose IV [39]. Cellulose I is associated with natural cellulose, easily found in nature. Cellulose II and cellulose III are by-products of the original cellulose, generally obtained through the regeneration of cellulose I. Finally, cellulose IV is obtained from cellulose III using procedures involving high temperatures and glycerol. The different crystal arrangements vary in terms of the characteristic attributes, such as hydrophilicity, mechanical potential, and stability performance [40].

Recently, cellulose-based exploration has been promoted due to a series of benefits, such as cost effectiveness, efficiency, physical and mechanical properties, the low degree of the environmental impact, exuberance, and capacity for nanoscale structure, among others [41]. A variety of technological innovations have been widely explored for the isolation of cellulose from lignocellulosic waste. The high interest has broken sustainability boundaries under the concept of biorefineries, because there is a wide spectrum of applications for cellulose-rich biomaterials or secondary bioproducts. Among the main industrial complexes that instigate research associated with cellulose are the food industry [42], textile industry [43], energy production [44], building and engineering industry [45], biomedicine [46], pharmaceuticals industry [47], adsorption [48], and wastewater treatment [49], among others.

#### Nanocellulose

Nanocellulose is a biopolymer originating from cellulose and occurring at the nanoscale, obtained mainly from marine and land plants, animals, and bacteria in four primary forms: CNC, cellulose nanofibers or nanofibrillated cellulose (NFC), microfibrillated cellulose (MFC), and microbial or bacterial nanocellulose (BNC) [50]. Nanocellulose is characterized as highly resistant fibers, with a diameter of less than 100 nm and a density of up to 1.6 g/cm^3^. A high abundance of hydroxyl functional groups can be easily adapted to express high performance [51]. Nanocellulose provides a highly modifiable surface, significant mechanical strength, high hydrophilicity, and biocompatibility [52]. During the hydrolytic process, the amorphous zone of the cellulose fibers is cleaved to form an extremely strong and crystalline nanoscale structure with a rod-like arrangement [53]. Most commonly seen, CNC features lengths of up to 100–300 nm and up to 5–50 nm in diameter, with a rich hydrogen bonding matrix, allowing for high voltage transfer. An NFC is commonly synthesized using chemical pretreatments and homogenization is carried out in high-pressure conditions [54]. NFCs constitute nanoscale fibrils, with a width between 2 and 60 nm, and are established from the agglomeration of cellulose chains, generated by hydrogen bonds, and comprise crystalline and amorphous zones, easily synthesized from the discharge of fibrils from microfiber bundles under strategies of mechanical fibrillation [55]. Furthermore, BNC consists of the application of microorganisms as primary sources of biopolymers, mainly due to the rapid microbial growth and high availability of the product. The literature indicates two dominant procedures to produce BNC based on microbial agents: static culture and agitated culture. Static culture refers to the accumulation of BNC forming a thick and whitish layer or cuticle. Agitated culture spontaneously produces cellulose in the culture medium, forming irregular agglomerates or suspended fibers [54].

CNCs are nanoparticles abundantly rich in fragments of the cellulose chain, rigorously ordered in a crystalline structure of up to 100 nm. CNCs indicate high thermal stability, in addition to a higher surface area and crystallinity compared to primitive cellulose [41]. NFCs are frequently produced by many mechanical procedures, such as milling/refining, high-pressure homogenization, ultrasound-assisted treatment, microwave, steam explosion, and microfluidization, and by a series of chemical processes, such as TEMPO oxidation, persulfate oxidation ammonium, carboxymethylation, and cationization [40].

**Figure 2:**
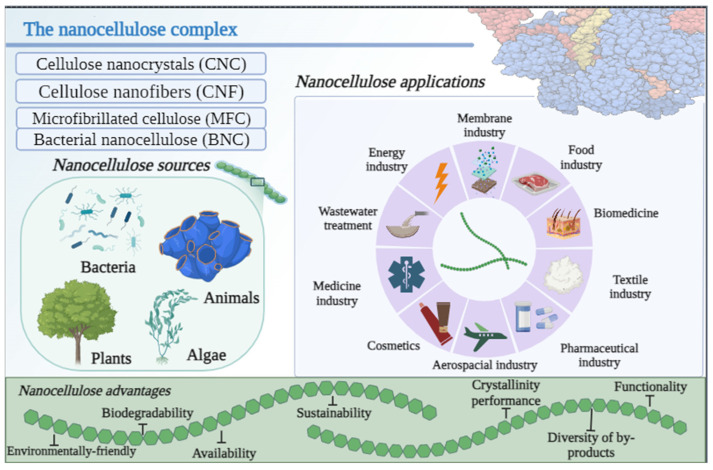
Main advantages and current applications of cellulose-based biomaterials and cellulose primary configurations.

The direct alteration of the surface of the cellulose nanoparticles allows access to the biopolymer for a variety of purposes (Figure 2). Modifications based on hydroxyl groups allow the improvement of the biomaterial and intensify its potential use. Chemical reactions involving oxidation and acetylation processes or the addition of functional materials, polymers, and functional groups on the surface of the nanogranules allow the surface properties of the nanocellulose to be improved and associate with different non-polar matrices or change its affinity with certain polar and non-polar molecules [56]. The use of nanocellulose has aroused extensive industrial interest and has shed light on a variety of operations, such as the paper industry [57], packaging [58], cosmetics [59], the pharmaceuticals industry [60], medicine [61], biomedicine [62], paints and coating [63], hydrogel synthesis [64], and filtrations [65]. Nanocellulose has two basic disadvantages, namely a high number of hydroxyl compounds, which causes strong and resistant interactions by hydrogen molecules between two bundles of nanofibrils, and high hydrophilicity, which does not allow its application for a variety of industrial purposes, such as coating paper or composites, for example, without inducing a prominent surface modification to degrade the number of hydroxyl interactions and to stimulate compatibility with several other matrices [66].

### 2.2. Lignin

Lignin is one of the most exuberant organic materials in nature and its content range is 15–30% in plants. However, these concentrations are variable depending on the type of biomass, plant characteristics, plant growth environment, and constitution of the cellulose wall [67]. Moreover, lignin is the only renewable aromatic polymer in nature [68]. Approximately 50 to 70 million tons of lignin are produced worldwide [69]. This panorama is directly associated with the widespread use of lignin as a source for the production of biofuels, since about 60 billion gallons of biofuels should be produced annually. Approximately 0.75 billion tons of biomass rich in lignin is required, indicating that the conversion of plant biomass will result in at least 0.225 billion tons of lignin as a by-product [70]. In the plant spectrum, lignin encompasses the free space between the cellulose and hemicellulose bands, establishing a highly resistant and rigid structure, whose purpose is to act in the performance of water and nutrient transport in the stems of plants [68]. The lignin matter is closely associated with the mechanical properties of the plant cell wall and the mechanical resistance provided by the biopolymer is significantly superior to the resistance provided by the cellulose content [71].

The inflexibility of lignin is highly influenced by the aromatic chains in the compounds in signapyl alcohol, *p*-coumaryl alcohol, and coniferyl alcohol. Furthermore, plant species with high lignin production have large amounts of lignin-synthesizing enzymes, such as phenylalanine ammonia lyase (PAL), caffeic acid *O*-methyltransferase (COMT), 4-coumarate coenzyme A ligase 3 (4CL_3_), cinnamyl alcohol dehydrogenase 2/7 (CAD2/7), cinnamoyl-CoA reductase 20 (CCR20), and cinnamate 4-hydroxylase (C_4_H) [72]. Lignin is also composed of three hydroxycinnamic alcohols, cetearyl alcohol, and mustard alcohol via ether associations, C-C chains, among others [33]. There is a significant diversity in distinct, highly polar chemical groups allocated in the structural complex of lignin, such as methoxyl, hydroxyl, carbonyl, and carboxyl, granting lignin high resistance to the action of enzymes, chemical solvents, or water hydrolysis [20].

Lignin acts as a carrier of fundamental materials, such as water and nutritional substances, and as a component of structural support for plant organs, arranging the matrix that also composes cellulose and hemicellulose in the complex [73]. The lignin content in the plant may vary with the species and the morphological organ, since there is a diversity in the scientific investigations that have indicated different concentrations of lignin in different organs of the same plant [74,75,76]. The high accessibility and ease of obtaining lignin from natural sources is key to a wide range of industrial applications, from adsorbent materials to biofuels and power generation [77]. The sustainable footprint of lignin provides the basis for the synthesis of biomaterials that convert the uncontrolled use of chemical resources to the production of electricity [78]. The spectrum of direct applications of lignin includes the engineering industry [79], biomedicine and biotechnology [80], medicine [81], biopesticides and biofertilizers [82], wastewater treatment [83], biofuels [84], adsorbents [85], carbon fibers [86], adhesives [87], dispersants [88], anti-UV filters [89], and the pharmaceuticals industry [90].

## 3. Modification and Characterization of Cellulose

Cellulose is widely obtained from lignocellulose-rich materials, bacteria, marine animals, and algae [91]. With the intensification of sustainable assertions in recent years, the exploration of polymers of natural origin has gained attention, which directly reflects the exploration of technological strategies and processes that involve the modification of these materials to enhance performance. The structural modification of the cellulose surface aims to reduce the high hydrophilicity of biomaterials, as well as to intensify the rupture of the long chain of hydroxyl groups that sustain the material. To improve treatment performance, it offers appropriate cost effectiveness and generates bioproducts in an environmentally friendly context. Pretreatments involving cellulose materials can be of enzymatic origin or TEMPO (2,2,6,6-tetramethylpiperidine-1-oxyl). These procedures aim to increase the reactivity of cellulose, especially in the transfiguration of hydroxyl groups into carboxylate groups [54]. The subtopics described below provide a better understanding of the processes involved in configuration changes in cellulose-based biomaterials.

### 3.1. Surface Modification

The surface arrangement of nanocellulose can be easily configured through the continuous action of surfactants rich in highly hydrophobic and hydrophilic groups, or the adsorptive process based on polyelectrolytes [66]. There is a diversity of surfactants, such as fluorosurfactants adherent to the cellulose structure, cationic surfactants, and polyelectrolyte compounds, adapting the hydrophobic potential and improving specific properties [54]. Alterations in the hydroxyl groups that form the surface structure of nanocellulose are appropriate to enhance the spectrum of action of these biopolymers, especially in association with other materials to configure the structural properties of nanocellulose and improve the field of affinity with highly polar and/or non-polar matrices [56]. The modification of the surface structure using the adsorption method is segmented into two main classifications: the polyelectrolyte method and specific groups aimed at the adsorption of some points. The polyelectrolyte method has high potential as it involves different polyelectrolytes with opposite charges and specific nanoparticles to adapt the desired properties to the nanoparticles, with ease of modification through the adsorption of the nanoparticles and CNFs [92].

### 3.2. Grafting

Graft polymerization is a cellulose modification strategy whose purpose is to stimulate highly resistant covalent bonds to generate a branched copolymer, without affecting the primary characteristics of the biomaterial [37]. The grafting procedure drastically reduces the interaction between solutes and unattractive aggregates with the cellulolytic surface, providing groups suitable for designing electrostatic repulsion from the membrane surface or enhancing hydrophilicity to enhance water-surface interactivity [93]. The grade of the grafted polymer directly affects the properties of the natural fiber, mainly the mechanical characteristics, elasticity, potential absorption, ion exchange competence, propensity for rupture of the resistant structure with extreme conditions of temperature and abrasion, and resistance [94].

Generally, the grafting procedure involves different mechanisms of action: (i) “grafting into” a step directly related to reactions between the functional groups of different polymers; (ii) “grafting from” refers to a polymer with functional groups that enhance the polymerization of vinylic monomers, in which the highly reactive sites belonging to the main chain are stimulated by chemical treatments or irradiation; and (iii) “grafting through” which implies the (co)polymerization of macromonomers [95]. Modification of the surface of cellulose by polymerization provides for the alteration of specific physical and chemical properties that may suit the desired purpose [96].

The effect of the grafting of cellulose in polylactide was evaluated after the synthesis of a series of cellulose ester–graft–polylactide (CeEs-g-PLA) copolymers. A series of CeEs-g-PLA copolymers was synthesized using one-pot reactions involving acylation and ring-opening polymerization. With the increasing degree of acyl group substitution, the copolymers presented enhanced thermal stability and thermoplasticity due to the intermolecular interactions between the acyl groups and polylactide sidechains. Therefore, the feed content of the acyl agent has a significant influence on the structural characteristics of the graft copolymer, because the acylation proceeds predominantly at the hydroxy groups in the cellulose backbone and, then, the PLA chains are grafted onto the remaining unreacted hydroxy groups [97].

Green biofilms with antimicrobial activity were developed from PLA and cyclic N-halamine 1-chloro-2,2,5,5-tetramethyl-4-imidazolidinone (MC) grafted microcrystalline cellulose (g-MCC) fibers. The grafting percentage was 10.24%. The grafting improved the compatibility between g-MCC and PLA, leading to an excellent dispersion of g-MCC in the film matrix, and a superior transparency of the g-MCC/PLA compared to that of the MCC/PLA films. The enhanced compatibility of the g-MCC/PLA films produced better mechanical properties, including the mechanical strength, elongation at break and initial modulus than those of both the MCC/PLA and MC/PLA composites. The oxidative chlorine of g-MCC/PLA was highly stable compared to that of MC/PLA films, providing long-term antimicrobial activity [98].

Incorporating the surface-grafted cellulose nanocrystals (CNCs) with enantiomeric polylactide (PLLA or PDLA) was presented as an effective and sustainable way to modify PLLA. The CNCs with identical content and length of PLLA and PDLA were prepared and blended with PLLA. The rheological properties of PLLA/CNC-g-D are improved, indicating that the stereocomplexation can improve the interfacial strength as compared with the conventional van der Waals force in PLLA/CNC-g-L. The matrix crystallizes at a higher rate in PLLA/CNC-g-L than PLLA/CNC-g-D. PLLA/CNC-g-L15 reached its half crystallinity in 8.26 min, while a longer period of 13.41 min was required for PLLA/CNC-g-D15. The formation of low content sc-PLA at the interface may restrict the diffusion of PLLA, but contribute less to generate crystalline nuclei, which synergistically leads to the retarded crystallization kinetics in PLLA/CNC-g-D [99].

### 3.3. Chemical Procedures

Chemical-based modification procedures involve changes in the basic properties of cellulose, such as the hydrophilic or hydrophobic potential, elasticity, water absorption, adsorptive or ion exchange performance, and resistance to adversity. The dominant basic chemical modification strategies for cellulose are esterification, etherification, halogenations, oxidation, and treatment with alkaline compounds [54]. Changes in the nanocellulose complex significantly increase the degradability and biocompatibility of the biopolymer with other biomaterials [53]. Furthermore, considering the low cost–benefit and process efficiency, Table 1 indicates the main segments and pretreatments for modifying the pulp structure, from specific chemical methods to mechanical base changes. The procedures increase the cellulolytic reactivity and enhance the conversion of compounds into desired functional groups to adapt to promising characteristics and properties.

### 3.4. Other Treatments

Furthermore, the diversity of viable alternatives has been applied to biowaste treatment. These strategies concentrate techniques of mechanical and/or thermal and chemical activities to alter the physicochemical properties of the feedstocks. Among the mechanical and physical methods, the drying method and the milling strategy are valid alternatives that have been widely explored. The drying method is extremely necessary for preparing the raw material before applying other pretreatment strategies, especially for eliminating moisture from the material, which improves process efficiency and requires lower temperature and calorific value [100]. Cellulose drying conditions directly influence its dissolution and some studies have led to a parameterization of conditions to optimize the cellulose dissolution process [95]. The drying process can be conducted by oven drying and/or freeze drying, hot pressing, and supercritical drying with CO_2_. Furthermore, the drying procedure or wetting/drying cycles, called hornification, provide higher dimensional stability and less material degradation through increases in molecular packing. This procedure can be controlled, for example, by the time and/or number of cycles and drying requirements [101].

Among the mechanical methods, strategies aimed at reducing the particle size and increasing the contact area between the solid matrix and the solvent are widely applied. The milling strategy involves the effectiveness of the mechanical and thermal effects to redesign the fiber matrix and provide a wide spectrum of applications for the biomaterials, based on the adjustment of high pressure, collision, and absorption, in addition to a significant increase in temperature [102]. Additionally, the milling procedure is an extremely efficient strategy for modifying the crystalline structure of cellulose, as it enables the optimization of cellulose hydrolysis, interrupting the crystallinity (cellulose I) of native cellulose through increased contact with acid by cellulose [103].

According to physiochemical methods, they are the most common alternatives, mainly due to the modifications in the properties of the material, as well as the increase in intermolecular interactions. These methods involve steam explosion, wet oxidation, liquid hot water (LHW), and microwave-assisted and ultrasound-assisted extractions, and have been widely explored due to the high rupturing of the lignocellulose complex and minimization of the crystallinity of the cellulose. Accordingly, the steam explosion process is an environmentally viable strategy to modify cellulose fibers through the intensification of fibrillation, providing the synthesis of nanofibers [104]. Furthermore, the steam explosion procedure promotes the rupture of lignocellulosic biomass components by steam heating, shear forces, and hydrolysis of glycosidic bonds by the organic acid formed during the process. The steam explosion procedure facilitates the rupture of lignocellulosic structures, promoting the modification of the physical properties of the material (specific surface area, water retention capacity, color, etc.) and increasing the rate of enzymatic hydrolysis of the cellulose components [105].

Wet oxidation is an interesting alternative applied to the functionalization of cellulose because the process results in products with different structures and properties depending on the substrate, reagents, reaction parameters, and medium. The strategy provides new, high-performance materials based on cellulose, with the possibility of a variety of applications [106]. The oxidation process involves changing the performance of nanofibrils, facilitating their dissolution in water. This scenario results in a high degree of cellulose processing, without requiring the use of chemical products [107]. Furthermore, pretreatment with liquid hot water (LHW) is an interesting strategy, since it does not involve the addition of chemicals and has moderate process operating conditions. The procedure involves the application of water associated with an increase in temperature, drastically reducing the pH of the medium releasing carboxylic acids and intensifying the rupture of the structural matrix of the lignocellulosic biomass. Consequently, there is a significant increase in the accessible surface area, intensifying the action of the enzymes and the fermentation process [108].

Furthermore, microwave-assisted technology is a promising technique applied to lignocellulose-rich structure modification processes and extraction procedures. The alternative applies microwaves to significantly increase the temperature of the medium, reducing the reaction time, improving the process efficiency, and establishing uniform operating conditions, such as fast heating speed, uniform heating, and no temperature gradient occurrence [109]. In the hydrolysis processes, the microwave-assisted treatment significantly promotes the transformation of cellulose into C6 molecules with high selectivity. High-temperature conditions act positively on hydrolysis performance, since the microwave-assisted process allows superior operating conditions compared to conventional hydrothermal systems. It was pointed out that high temperatures promoted an intensification of the association at the molecular level between the microwaves and cellulose (through the primary alcohol groups, –CH_2_OH groups), redirecting the energy to the surrounding molecular structure to initiate the cleavage of polysaccharide chains [110].

Ultrasound-assisted technology has been indicated as an efficient strategy in the extraction and rupture processes of the lignocellulosic complex. The energy intensity of the process increases the mass transfer of the biomass components to the extraction solution and, under established conditions, causes the acoustic cavitation process, in which the waves produced by the equipment propagate in expansion and compression cycles. Large amounts of microbubbles are formed and collide with strong motion. The friction between the microbubbles releases a significant amount of energy in the configuration of shock waves, which come into contact with the material rich in lignocellulose and promote its disintegration, facilitating the extraction and modification processes [111]. The hydrodynamic forces produced lead to the defibrillation of the biomass, which may be pure cellulose, microcrystalline cellulose, or other components of interest. The direct rupture of the biomass promotes the formation of filament aggregates with different sizes. The performance of the process depends directly on the characteristics of the material, since the ultrasonic bath acts on the crystalline structure of cellulose in different ways, based on the type of biomass, operating conditions, concentration of lignocellulose, and degree of crystallinity [112].

On the other hand, the use of organic solvents is still one of the main alternatives adopted as a pretreatment. The replacement of the primary hydroxyl groups in cellulose by other molecules results in the intensification of the diversity of chemical reactions, in addition to contributing to an increase in grafting efficiency and the performance of functional groups for structural modifications. The main chemical reaction alternatives applied for the structural modification of cellulose are esterification, oxidation/amidation, and silanization. Esterification is generally carried out by an acylation process with carboxylic acid anhydride and 4-dimethylaminopyridine or strong acid as a catalyst. The oxidation process involves distinct C6 hydroxyl groups under moderate aqueous conditions; in addition to modifying the biopolymers and causing strong bonds at one end and adapting them with specific functional groups at the other to adapt to the matrix [113]. The application of silane is widely carried out, since the process intensifies the interfacial interaction between the hydroxyl groups of cellulose. The silanol agent is produced and can react with the hydroxyl groups of cellulose or condense on cellulose surfaces since they have the same reactive groups (-OH). Furthermore, thermal treatments can allow condensation between the OH groups of hydrolyzed silanes and cellulose, assuming chemical modification [114]. Nevertheless, these materials are highly harmful and their recovery after the extraction procedure requires additional steps, which results in higher process complexity and increased cost–benefit [115]. The continued use of solvents in pretreatment procedures is still inevitable, mainly due to the high proportion of solid dissolution, mass and heat transfer, viscosity reduction, and effectiveness in the separation and purification operation [116].

**Table 1:** Current advantages and limitations to the main cellulose-based modification processes.

Modification Methods	Process Methods	Advantages	Drawbacks	References
Surface adsorption	– Plasma – Photochemistry – Radiation – Enzymes	– Hydrophilicity – High efficiency – Environmentally friendly – Biocompatibility – Properties adjustment – Cellulose profile preservation – Biodegradability	– High moisture absorption – Uncontrolled di-isocyanate and cellulose reaction	[37,96,117]
Chemicals	– Carboxylic acid groups – Specific functional groups – Alkyne–acid associations – Carbonylation – Esterification – Acylation – Ionic liquids – Etherification	– Functionality – Viability for a variety of functional groups – Process agility – Efficiency – By-products generation – Structural durability – High adsorption potential	– Pollution rate – High costs – Recycling resistance – Low dispersibility – Purification necessity	[37,54,118]
Grafting	– Grafting to – Grafting from – Grafting through	– Versatility – Biocompatibility – Properties adaptability – Weight adjustment – Dispersity adjustment	– Homopolymer synthesis – High graft density – Degradation of cellulose complex – No generation of block copolymer grafts	[95,96,119,120]
Molecule chemical grafting	– Ionic transference – Esterification – Acetylation – Gaseous methods	– Cellulose structure improvement – High cellulose derivates production – Process conditions adjustment – Accessibility of the cellulose surface	– High complexity in ester bond production – No total cellulose dissolution – Toxicity – Harsh reactant conditions	[37,54,66,120]

## 4. Modification and Characterization of Lignin

Lignin is the second most abundant biopolymer in nature, with a highly resilient structure and strong antioxidant activity. The molecular design of lignin indicates a significant concentration of functional groups, with easy alteration of properties based on chemical modification procedures [121]. The concentration and design of the lignin matrix varies depending on the type of biomass and lignocellulose content [122]. The golden age of exploring highly sustainable energy sources based on the use of materials rich in lignocellulose comes from strategies for a diversity of applications, such as the mass production of biofuels and other biochemical products to satisfy energy demand. Some essential plant materials from widely cultivated crops, such as sugar cane, corn, and sorghum, are promising for processes involving the synthesis of first-generation biofuels and chemical products of interest [33]. Under biorefinery concepts, the type of biomass is also strongly influenced by local characteristics, such as agricultural management, climate performance, and raw material availability. Since the bioeconomy approach has emerged as a strategy faithfully associated with the valorization of residues of plant origin, the requirement for natural biopolymers has fueled interest in technological alternatives and methods associated with the modification of lignin [123].

Considering that the structure of lignin is rich in a diversity of active groups, lignin can react chemically from different aspects, such as halogenation, nitration, phenylation, graft copolymerization, alkylation, dealkylation, sulfomethylation, acylation, ammonization, esterification, and hydrogenolysis. Furthermore, lignin has satisfactory compatibility with other biopolymers or natural fibers due to its hydrophilic nature, which establishes the application of lignin polar groups as agents to increase compatibility with essentially hydrophobic polymers. Furthermore, cross-linking with other polymers is desirable from the application of their hydroxyl groups to give rise to new materials, such as aromatic chemicals and bio-based polymeric materials [124].

One of the main methods of modifying lignin consists of the esterification of the biopolymer in reactions involving carboxylic acids, anhydrides, and acid chlorides. In this case, the modification of lignin by esterification reaction causes significant changes in its properties, such as better UV absorption, altered thermal stability, higher compatibility with the matrix, improved mechanical properties, better dimensional stability, improved hydrophobicity, and higher resistance to microbial decomposition [125]. Other surface modification strategies, such as conductive polymer coating, gold spray coating, and metal oxide coating, have received attention, due to the tunable physicochemical properties that have a wide range of uses, such as energy storage, sensors, and adsorption propensity [126]. Other methods involve physical modification techniques, which do not involve reactions between the functional groups present in lignin, but explore physical strategies that promote new and distinct properties of the modified material. Among these techniques, the application of gamma irradiation, sorption of metal ions, and plasma treatment are excellent exemplifications. These alternatives cause strong variations in the morphology of lignin, ease the rupture of the rigid matrix, and cause alterations in the surface characteristics of the material.

## 5. Manufacturing Technology

### 5.1. Extrusion Technology

An extrusion machine can be a single or twin-screw machine. A twin-screw extruder offers better efficiency results by reducing the melting and mixing time. Three important aspects related to extrusion technology are polymer melting, solids transport, and melt flow, which are controlled by computer models. These extrusion models are limited to pure polymers, so when making a composite there are difficulties in the fluidity of the reinforcing material [122]. However, a model called global GSEM has recently been developed for the extrusion of reinforced polymers in single-screw extruders with flood and starvation feeding, where starvation feeding has advantages to melting, less agglomeration, and better compound mixing [127].

Extrusion is a technology that is generally used as a preliminary step to injection molding. In extrusion, the matrix and the reinforcement are mixed to form granules, which are then laminated with injection technology [23,128].

There is research using extrusion as a pre-injection stage using vegetable-based materials. Mainly when producing pellets, this is the case in the study of thermoplastic starch and polylactic acid with tannins to delay biodegradation [129], to evaluate compatibilizers between polylactic acid and thermoplastic starch [130], with residues of soy, polyvinyl alcohol, and starch [131], or the use of bagasse cassava with polylactic acid for the production of tubes for seedlings [132].

### 5.2. Injection Molding (IM)

The injection molding process has four relevant phases: plasticization, injection, cooling, and ejection [133]. During the first phase, the material is inserted into the barrel through a hopper and is melted using a rotating screw and internal heating units. Once the material is melted, it continues to the next phase, where the material is injected into the mold at a set speed and pressure parameters. For this, the screw is shifted to the front to avoid pressure variation and backward movement of the material in the barrel or deformation of the material. After this, the molded part goes to the cooling phase where the pressure and temperature decrease. This phase ends when the material solidifies. Finally, in the ejection, the part is removed by opening the mold [134,135,136]. IM technology demands high-energy consumption during processing [137]. The cooling phase is the most time-consuming stage in the cycle, taking between 50% to 80% of the cycle, so it is the stage that consumes the most energy [138]. As a result, there are more and more studies on improving energy efficiency at different stages of the process [139,140,141,142].

#### Parts of an Injection Molding Machine

The fundamental aspects to consider for IM are the machine specifications and the material to be used. Optimizing these aspects can ensure the reduction of defects and the quality of the final products [143]. An injection molding machine has a motor. It can be an AC motor or a hydraulic motor, with the hydraulic motor being the most used due to its excellent characteristics, such as less force required to start the movement and less overload on the rotating screw [134]. Then, it has a hopper to receive and store the material until it passes into the barrel to be melted, with the help of the heating units and the rotary screw. As the pellets are moved forward by the screw, they gradually melt, and are entirely molten by the time they reach the front of the barrel. In this part, there are temperature control sensors for the resistors. To complete the injection unit parts, we have the non-return check valve and the nozzle that contacts the mold and through which the material is injected. In the clamping unit, there are the fixed platen and the mobile platen that hold the mold [22,144].

**Figure 3:**
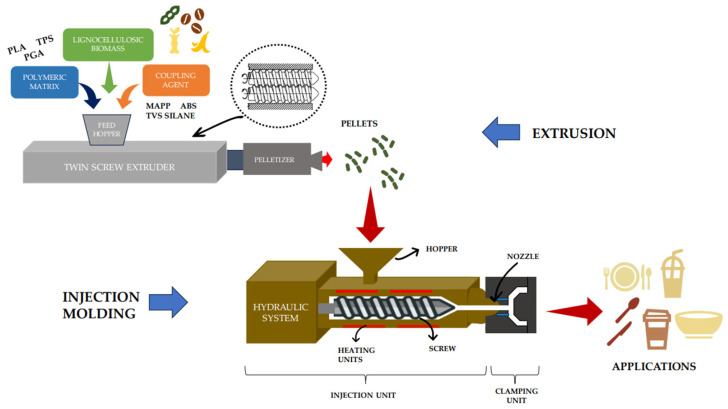
Extrusion and injection molding to produce composite-based parts.

Additionally, water is involved in the injection process. The plastic, which has the consistency of warm honey, is too viscous to flow through the narrow vents. To speed up the plastic’s solidification, coolant, typically water, flows through channels inside the mold just beneath the surface of the interior. After the injected part solidifies, the mold opens. As the mold opens, the volume increases without introducing air, which creates tremendous suction that holds the mold together [145]. The extrusion and injection molding process described is illustrated in the following Figure 3.

### 5.3. Materials

The materials used for IM can be thermoplastic or thermosetting. Some of the polymers used are PA 6, PC, PE-HD, PE-LD, PP, and PS, although there is an extensive variety [146]. Currently, due to the growing interest in biodegradable compounds, petroleum-derived polymers are being replaced by biopolymers obtained naturally or synthetically, such as PLA, TPS, PGA, PHB, PLLA, etc. To select the most suitable polymer for IM, it is important to consider some of the relevant inherent properties, such as strength, flexibility, toughness, thermoresistance, and cost [147].

### 5.4. Polymer Composites: Issues, Challenges, and Progress

#### 5.4.1. Cellulose and Hemicellulose Used in Injection Molding

Cellulose and hemicellulose in injection molding are generally used as reinforcing materials in bonding to a matrix polymer. The mechanical, thermal, and morphological properties of injection molded reinforced composites are the focus of research and discussion, since these properties are parameters to evaluate the improvements that the addition of lignocellulose to the polymer matrix can provide. The parameters of reinforced polymers are mainly linked to the pretreatment of the fiber, the percentage of the filler to be used, the dispersion of the fibers in the matrix, the technology used, the fiber length, and the properties of the matrices [148].

The compatibility between the matrix and the reinforcement represents a challenge due to the hydrophilic behavior of the filler and the hydrophobic behavior of the matrix, resulting in fiber agglomeration [149]. For this reason, coupling agents that act both in the matrix and the filler are currently used to improve the adhesion, heat resistance, and mechanical properties of the composite. The most used coupling agents are epoxy and maleic groups and glycidyl methacrylate because of their favorable compatibility [150,151,152]. One of the agents most widely used as a compatibilizer is maleic anhydride grafted polypropylene (MAPP), as it provides good adhesion when a polypropylene matrix is used [153,154]. The correct adhesion between the fiber and the matrix will integrate the fiber-dependent strength and modulus and the matrix-dependent thermal stability.

For the formation of parts by extrusion and IM using biomass as reinforcement, it is important to consider the processing temperature. Lignocellulose has two zones where its main components are lost, between 200–250 °C where amorphous cellulose and hemicellulose are degraded, and between 360–540 °C where lignin is degraded [150,155,156,157]. This parameter can affect the tensile strength and stiffness of the obtained product [158]. Organoleptic characteristics, such as odor and color, are also affected by high temperatures, even though cellulose has a high thermal resistance. Hemicellulose, on the other hand, decomposes producing an inappropriate odor, however, this can be minimized with odor attenuators [159]. The color of the molded part can undergo variations depending on the reinforcement material used, such as turning a dark brown color due to the Maillard reaction [160].

In injection molding and extrusion, the reinforcement material and the matrix material influence the rheological, mechanical, and thermal characteristics. Overfilling can reduce the contact between the reinforcement surfaces and the matrix due to the lack of available contact surfaces in the matrix, which will affect the mechanical properties by preventing energy absorption and enhancement of the matrix polymer [161]. Regarding the rheological properties, the increase in filler material does not significantly affect the viscosity or melt temperature [153], but it can generate an increase in pressure, which can cause clogging of the nozzle during injection and generate defective parts [162]. In some cases, the coupling agent has been shown to reduce viscosity by providing lubrication, which may reduce the pressure required during injection [163]. In the study on a composite reinforced with coffee husk flour, they evidenced fractures in the rough surface due to the increase in filler [160]. Increased filler and poor adhesion can affect the toughness of the composite and promote brittleness, as evidenced in tests conducted between linseed meal and PLA. However, this can be significantly reduced with the use of linseed derivatives, such as oil, which serve as a plasticizer during extrusion [164].

Extrusion is commonly used as a previous step to IM, used to make the blend of the reinforced composite. Hence, it is very important to try to optimize the parameters during this process. A failure related to the extrusion of fiber-reinforced polymers is breakage due to stress accumulation in the fibers. This is mainly due to the control of parameters through the extruder flow [165]. The size of the fibers and the shearing can also affect the mechanical properties since the adhesion between the compounds is reduced [151,166]. It reduces the surface interaction between the filler and the matrix, as well as overfilling, promoting agglomeration and a reduction of Young’s modulus [167]. The tensile modulus will increase as the fiber length increases [158]. One technique that has shown promising results in the processing of polymer composites before injection is solid-state extrusion (SSE), as it favors fiber distribution and dispersion [149].

#### 5.4.2. Lignin-Based Polymeric Materials

Due to its hydrophobicity and rigidity, lignin is of direct use, however, it requires hard work for its integration with a polymer matrix [168]; in addition to being incompatible with various aliphatic polyesters, such as PLA and PLC, impairing its mechanical properties [169]. In the evaluation of the addition of unmodified lignin extracted from tobacco in HDPE, it was found that the injection molding parameters are not affected and the dispersion using a single-screw extruder is adequate; however, the increase in lignin decreases the resistance to traction [170]. A coupling agent in lignin-reinforced composites, such as maleic anhydride grafting, can improve the tensile strength and ethylenebutylacrylate glycidylmethacrylate terpolymer (EBGMA) impact resistance. The combination of both can offer better results in terms of both characteristics [171]. More recent studies have seen advances in composites by extrusion and injection with hybrid components (pp/lignin/linen) using MAPP to ensure adhesion, obtaining improvements in stiffness and strength [172].

Regarding advances in extrusion and injection technologies, biobased polyethylene and kraft lignin processed using reactive extrusion with dicumyl peroxide (DCP) offer suitable results in terms of the mechanical properties and dispersion in lignin, thus being an effective and sustainable alternative [173]. Kraft lignin can also be used as a bio-coupling agent when modified by phenolation or glyoxalation, giving similar results to those obtained with maleic anhydride grafting concerning the mechanical properties [174].

## 6. Applications

In recent years, the number of biodegradable materials from different agroindustrial wastes and by-products has increased because of the need to replace the use of conventional petroleum-based plastics. In this context, developing biodegradable plastic (natural polymers or biopolymers) is necessary to avoid recycling and environmental pollution issues. It also has several advantages, such as renewability and biodegradability, and can be part of sustainable consumption that minimally affects the environment [175]. This agroindustrial biomass may be directly incorporated into polymer matrices, reinforcing filler composites [175], or used as the source of particular compounds to modify the polymer materials [176].

Corn, wheat, rice, soybean straw, sugarcane bagasse, orange waste, coffee industry by-products (coffee husk, spent coffee grounds) [177], avocado seed flour [175], banana and pineapple wastes, cornhusk, malt bagasse, and a diversity of residues are used in polymer matrices (polyolefins, low-density polyethylene, polyhydroxybutyrate, high-density polyethylene, and polypropylene). They are used for the manufacturing of natural fiber composites (NFCs), mainly to promote mechanical reinforcement and thermal or acoustic insulation [178], since they have thermal conductivity similar to these materials. They have already been implemented in the automotive, aerospace, and defense industries, where innovations are being made [179]. It uses trays prepared by thermopressing in a compression molding machine to fabricate biodegradable trays for semi-rigid packaging [180].

In agricultural, agroindustrial wastes, such as corn and wheat-waste flour, sunflower seed husks, rice husks, yerba mate waste, and cellulose paper, are used in the development of biodegradable and compostable pots for seedling growth containers molded from the obtained thermocompressed sheets using a mold with the specified dimensions [181]. In civil construction, studies have been developed on the application of vegetable fibers as reinforcement in cement-based composites and particleboards for building construction and infrastructure, for applications as ceilings and as structural components [182].

Applications of polymeric materials from agroindustrial biomass include household goods, sports equipment, musical instruments, toys, office supplies, flexible cards, and within the automotive industry in the form of pellets by injection molding and extrusion [67,158]. Regarding the food industry, it has been used extensively for the formulation of food packaging and containers, such as trays, plates, bags, cups, and lids. In the food services sector, it has been used to produce spoons, forks, knives, and drinking straws, as shown in Table 2.

The use of PLA in combination with cassava bagasse accelerates biodegradation faster, its use as seedling tubes increases the phosphorus content of the soil [132], cassava bagasse also increases the tensile strength, the modulus of elasticity, and lowers the water absorption capacity [201]. Besides, their use as lignocellulosic nanofibers, in combination with cassava starch, they obtain good intermolecular interaction and barrier properties, which can be applied in food packaging [202]. Moreover, cassava could also be used as a matrix. One study used cassava starch with glycerol and water, this mixture in combination with acerola improves the elongation at break, but reduces the mechanical properties and elasticity due to its high sugar content, while the mixture with added grape residues, improves the mechanical properties and elasticity [197]. Furthermore, grape pomace extract as an antimicrobial additive in bactericidal isotactic polypropylene shows low water vapor permeability [203]. PLA composites in combination with wheat straw were also developed, demonstrating rapid crystallization for a shorter molding time, and increased flexural modulus, and water permeability for packaging [200]. In addition, the use of ultrafine wheat fiber, blended with PHBV, improves the water vapor transfer rate, favoring its use in fresh produce packaging [204]. On the other hand, lignocellulosic nanofibers developed from wheat straw, blended with PLA and Ecoflex^®^, resulted in high water vapor permeability and antioxidant capacity for lettuce packaging [205]. Other research, using raw wheat bran composites and PBS, showed a noticeable impact on the rate of decomposition in an accelerated ageing environment [206]. PLA with mango by-products (20% tegument) achieves good mechanical properties, such as an elastic modulus up to 38% by IM, fiber provides higher stiffness, applicable for rigid packaging [187]. Mango seed and its use as a flour, mixed with glycerol, increases the mechanical and barrier properties, and has good antioxidant capacity [207,208]. In addition, the development of bioPP/mango peel flour for wood product applications and compatibilized with an itaconic acid copolymer, results in increased Shore D hardness, tensile strength, and an increased fracture toughness of 29.69% [185]. PLA with coffee grounds (SCG) by IM and compatible with oligomers, possesses high thermal stability, tensile strength, and elongation at a break of 39.6%, due to its lipid content. These were applicable for utensils and tableware [183]. Another study also incorporated the use of PLA and SCG through the blown extrusion process to produce biocomposite films, showing that the elongation at break increases with increasing SCG, while the tensile strength and hardness decrease [199]. This is because oil extraction from SCG increases the flexibility in films [208], and its incorporation also increases the content of antioxidants and microbial activity [209]. The use of coffee waste continues to increase, such as the production of coffee capsules based on coffee silverskin (tegument covering the endosperm) with PHBV copolymers by IM, resulting in a low breaking strength but an increase in the elastic modulus and crystallinity [192]. Food packaging was also made from coffee husks, HDPE, and ABS, as a result of the increased tensile modulus and tensile strength [195].

Yerba mate waste at 20% by weight, blended with PP and HDPE, showed good modulus and tensile strength, viable for wood composites [188]. Likewise, the use of yerba mate residue with PLA increases the flexibility and preserves the antioxidant properties, applicable in films [210]. Bean waste can also be applied in film making, its use at 30% by weight, in combination with PBSA/PHBV, increases the modulus of elasticity and decreases the tensile strength, applicable in the production of films [166]. Banana fiber and Mater-Bi^®^ were used in the creation of biodegradable bags, presenting greater strength and flexibility due to the fibers. In addition, the ripening of bananas is delayed by 1 to 2 weeks [189]. On the other hand, banana fibers with PVA increase the tensile strength and minimum water absorption for film making [211]. Similarly, banana fiber (especially canary fiber) has a higher tensile strength and modulus of elasticity compared to other fibers, such as sisal, jute, flax, cotton, and coconut [212].

Walnut shells were combined with PP by means of IM for panel, board, and plywood production, demonstrating that using PP and MAPP as a bonding precursor provides stiffness and thermal stability [184]. In the production of wood-based panels, walnut shells could be added up to 20% in order to fulfil their mechanical properties [213]. In addition, it has been shown that nut shells are very fragile in combination with PLA, so alkaline treatments are used [214] or plasticizers, for example, epoxidized oils are used [215]. Another study used PLA with durian skin fiber and additionally epoxidized palm oil, which resulted in improved processability and energy reduction, applied in biodegradable packaging [198]. Moreover, sheep wool fibers were used as reinforcement for PLA plasticized with maleinized linseed oil, resulting in poor tensile properties, but an increase in the modulus of elasticity and the elongation at break [193]. The increased use of wool fibers does not generate good adhesion in the polymer matrix, which decreases the tensile forces [216]. As a solution, silane treatment generates good compatibility and adhesion to wool fibers, increasing the mechanical properties [217]. Recycled cotton fiber waste has been used with bio-PET by IM and showed poor mechanical properties, such as tensile strength, due to different polarities; however, they have a high modulus of elasticity and hardness, applicable for rigid packaging [191]. On the other hand, films with cotton and elastane residues through dissolution and regeneration, obtain high transparency and good tensile strength, applicable for packaging materials [218]. Another study used Carbocal^®^ (sugar-beet residue) with LLDPE, resulting in stiffer composites; the thermal resistance and modulus of elasticity increased by 150% with the use of 50% Carbocal^®^ [194]. The use of sugar beet with PVA increases the mechanical properties and water resistance for the formation of biodegradable films [219].

## 7. Concluding Remarks and Future Trends

Advances in polymeric materials derived from agroindustrial biomass using extrusion and injection molding techniques present a promising avenue for sustainable material development. Biomass offers renewable, eco-friendly feedstock for biomaterial production, reducing the environmental impact and providing cost savings. The modification of cellulose, achieved through surface modification, grafting, chemical procedures, and molecule chemical grafting, improves the properties and versatility of these materials. However, more studies are needed to optimize the modification and processing techniques, improve material compatibility, and explore new applications. Extrusion technology melts, mixes, and homogenizes the composites to form granules. Twin-screw extrusion is suggested as it provides greater efficiency in the process, followed by the use of injection molding technology to obtain the desired shape. The parameters in extrusion and injection molding should be optimized, as they will depend on its shear and composition. The fiber size is of vital importance because it can reduce the mechanical properties, such as Young’s modulus of elasticity, which is why solid-state extrusion is recommended, as it favors a better distribution of the fibers. In addition, to overcome the differences in polarity between the matrix and the reinforcements, coupling agents are used to improve the adhesion, mechanical properties, and thermal stability of the reinforcements. Continued advancements in this field will contribute to the transition towards more eco-friendly and resource-efficient material solutions. Extrusion and injection molding techniques offer remarkable advantages as a result of their application in multiple industrial sectors, such as the automotive industry, textiles, pharmaceuticals, the biomedical industry, various packaging applications, and many others.

According to this review, it is evident that the trend is for biodegradable materials applied to the area of agriculture and food. The most common biodegradable polymer used is polylactic acid. Depending on the application and rigidity of the material, extrusion or injection is preferable. For example, more flexible materials, such as bags, are better for extrusion and more rigid materials, such as utensils or tableware, are preferable.

## Figures and Tables

**Table 2:** Applications of compounds.

Application	Matrix	Reinforcing Material	Processing Method	Coupling Agent	Improved Properties	Ref.
Seedling tubes	PLA	Cassava bagasse	Extrusion and IM	-	Increased biodegradability	[132]
Food-serving utensils and tableware	PLA	Spent coffee grounds	Extrusion and IM	Oligomers of lactic acid (OLAs)	Tensile strength ductility and thermal stability	[183]
Masking panels, fiberboards, and plywood	PP	Walnut shells	Twin-screw extrusion and IM	MAPP	Thermal stability	[184]
Floors, doors, and furniture	BioPP	Mango peel flour	Reactive extrusion and IM	PP-g-IA and DCP	Elongation at break and thermal stability	[185]
Bio packaging materials, and food films	PBSA/PHBV	Faba bean stems and pods	Twin-screw extrusion and IM	-	Mechanical and barrier	[166]
Pellets (for packaging and disposable utensils)	PBS	Raw wheat bran	Extrusion	-	Increased biodegradability	[186]
Rigid food packaging	PLA	Mango seeds	Extrusion and IM	-	Mechanical and barrier	[187]
Thermoplastic composite products	HDPE	Yerba mate	Extrusion and IM	MAPE	Tensile strength and modulus	[188]
Protective bags for banana fruits	Mater-Bi	Banana fiber	Twin-screw extrusion and film blowing/IM	-	Flexibility and mechanical properties	[189]
Bio composites	PP	Bagasse cane	Twin-screw extrusion and IM	SEBS-g-MA	Yung’s modulus, tensile strength, and hardness	[190]
Rigid food packaging	Bio PET	Recycled cotton fibers	Twin-screw extrusion and IM	-	Elasticity, hardness, and thermal stability	[191]
Coffee capsules	PHBV/ATBC/CaCO_3_	Coffee silverskin	Melt extrusion and IM	-	Elastic modulus, crystallinity, and biodegradability	[192]
Industrial materials	PLA/MLO	Sheep wool fibers	Extrusion and IM	TVS silane	Matrix/reinforcement interaction	[193]
Packaging products	LLDPE	Carbocal	Extrusion and IM	-	Mechanical and rheological	[194]
Food packaging and industrial applications	HDPE	Coffee husk	Extrusion and IM	Acrilonitrilo butadieno estireno (ABS)	Tensile modulus, tensile strength, and melting temperature	[195]
Food stretch film, food shrink film, and bags of fruit	PE	Sour cherry shell powder	Single-screw extrusion with blowing die film	Maleic anhydride polyethylene	Mechanical and moisture absorption	[196]
Flexible bioactive packaging	Starch/glycerol/water	Acerola residue	Extrusion and IM	-	Antioxidant characteristics	[197]
Rigid bioactive packaging	Starch/glycerol/water	Grape skin	Extrusion and IM	-	Antioxidant characteristic	[197]
Biodegradable food packaging	PLA	Durian skin fiber	Extrusion and IM	-	Biodegradability	[198]
Agricultural film products	PLA	Spent coffee grounds	Twin-screw extruder and blow film extrusion	-	Flow rate increasing and viscosity decreasing	[199]
Fruit and vegetable packaging	PLA	Wheat straw	Twin-screw extrusion and IM	-	Flexural modulus and mechanical and thermal performance	[200]

## Data Availability

Not applicable.
